# The Effects of Unilateral Pulvinar Damage in Humans on Reflexive Orienting and Filtering of Irrelevant Information

**DOI:** 10.1155/2002/917570

**Published:** 2002-01-01

**Authors:** Shai Danziger, Robert Ward, Vanessa Owen, Robert Rafal

**Affiliations:** ^1^ Ben Gurion University of the Negev Beer Sheva Israel; ^2^ University of Wales Bangor UK

**Keywords:** pulvinar, thalamus, attention, selection, interference, orienting

## Abstract

The effects of damage to the pulvinar nucleus of the thalamus in humans on reflexive orienting and selective attention were investigated. In a spatial orienting task three patients with unilateral pulvinar damage determined the location of a visual target that followed a cue that was not informative as to the targets location. Contralesional targets were responded to more slowly than ipsilesional targets. Also, at long cue target intervals patients responses to contralesional targets that appeared at previously cued locations were slower than to non-cued locations indicating that pulvinar damage does not affect inhibition of return. In the selective attention task two of the patients identified a target that appeared at one level of a global-local hierarchical stimulus while ignoring a distractor present at the other level. The distractor indicated either the same response as the target or a different response. Response times to targets in both visual fields were similar as were interference effects from the ignored distractors. These data indicate that engaging attention contralesionally is not impaired in discrimination tasks and that filtering of irrelevant information was not impaired contralesionally.

## 1. Introduction

In a cluttered sensory environment successful control of behavior requires selective processing of goal-relevant information and suppression of irrelevant information. The process responsible for this selectivity has been named attention. On current views attentional processing is carried out by a distributed network of brain regions that includes both cortical areas such as the parietal cortex (PAC), and prefrontal cortex (PFC), and subcortical structures including the superior colliculus and the pulvinar nucleus of the thalamus [1,2]. The aim of this study was to investigate the contribution of the human pulvinar to reflexive orienting and selective filtering of irrelevant information.

Despite a growing body of research on the role of the pulvinar in attention from single cell recording [3] and reversible lesion studies in non-human primates [4, 5], the pulvinar's role in attention in humans has been examined in only a few studies that have yielded somewhat inconsistent results [6–8]. For example, while Rafal and Posner [8] reported impaired contralesional orienting following pulvinar damage in a spatial cueing task that required target detection, Danziger et al. [6] found normal contralesional orienting following pulvinar damage in a response competition task that required target identification. Inconsistencies have also been found concerning the pulvinar's role in filtering irrelevant information. Based on positron emission tomography (PET) data that showed selective pulvinar activation under conditions that required identification of a target in the presence of distracting information, Laberge and Buchsbaum [7] proposed that the pulvinar contributes to filtering irrelevant information. On this view pulvinar damage should produce a deficit in filter-

ISSN 0953-4180/01/02/$8.00 © 2001/2002 – IOS Press. All rights reserved ing contralesional distractors. Danziger et al. [6] failed to confirm this prediction finding equivalent flanker interference from contralesional and ipsilesional flankers in their response competition study of patients with unilateral pulvinar damage. The existing inconsistencies in the literature were the impetus for the present study that had two primary aims. First, to determine whether pulvinar damage in humans affects reflexive spatial orienting in a manner consistent with that suggested by Rafal and Posner [8] for voluntary orienting and second, to further test the notion that the pulvinar contributes to filtering irrelevant information.

## 2. Series 1: Reflexive orienting

Rafal and Posner [8] used a spatial cueing paradigm to examine the pulvinar's role in spatial orienting. In this experiment patients with unilateral pulvinar damage were asked to detect as quickly as possible a target stimulus that appeared either to the right or left of fixation. On each trial the target temporally followed a cue that appeared at one of the two possible target locations. To induce voluntary orienting of attention towards the cued location the target appeared at the cued location on 80% of the trials. At cue target intervals that ranged from 50 to 1000 milliseconds (ms) patients' performance was characterised by longer reaction times to contralesional targets whether attention had been cued contralesionally or ipsilesionally. From this finding Rafal and Posner [8] suggested that the pulvinar contributes to engaging attention contralesionally. In their framework of spatial orienting, the engage operation was distinguished from a move operation thought to be mediated by midbrain structures and a disengage operation thought to be under parietal control [2].

The aim of Experiment 1 was to extend the investigation of voluntary spatial orienting in patients with pulvinar damage to the domain of reflexive orienting. Previous studies using spatial orienting tasks have demonstrated dissociations between these two modes of orienting. For example, while voluntary orienting to cues that correctly indicate the target location on a high percentage of trials is associated with sustained benefits in performance at cued locations, reflexive orienting to uninformative cues is associated with faster responses to targets at cued locations at short cue-target stimulus onset asynchronies (SOAs) and slower responses to such targets at long cue-target intervals [9–11]. It has been suggested that this latter effect, known as inhibition of return (IOR), aids visual search by producing a bias in orienting to novel locations [12]. A study by Sapir and colleagues [13] in a patient with a unilateral superior colliculus lesion who failed to show contralesional IOR suggests that IOR is generated by the superior colliculus. Because of the anatomical links between the superior colliculus and the pulvinar one question of interest was whether pulvinar damage would affect contralesional IOR.

### *2.1. Methods*

#### *2.1.1. Patients*

All three patients in this study had been neurologically intact prior to sustaining hypertensive haemorrhages centered in the posterior thalamus. In all cases the patients had returned to independent living with some residual motor or sensory deficits, but had no mental symptoms compromising functions of daily life and social interactions. Participation in the experiments began 2–5 months after the initial haemorrhage and after oral and written consent was obtained. The experimental protocol for experimental testing and MRI scans was approved by the North Wales NHS Trust Ethics Committee. MRI scans were obtained at the time of testing and included T1 weighted 1mm sections in axial, coronal and saggital planes. In all patients the residual lesion involved the posterior lateral nucleus of the thalamus and extended to involve the pulvinar nucleus. The entire pulvinar appeared to be destroyed in patient SM. Most of the lateral pulvinar was destroyed in patient GJ. For patient TN, only the most anterior and superior parts of the pulvinar were destroyed. Representative MRI sections are shown in Fig. 1.

T.N is a 54 year old, right handed, hypertensive woman who suffered a haematoma centered in the right thalamus in June 1999, 5 months prior to testing. Examination during testing revealed weakness and hyperactive reflexes in the right arm and leg. She could move her fingers but could not oppose the thumb to the 5th finger. Pin sensation was present but less intense on the right. Temperature sensation seemed equivalent bilaterally. Position sense was absent in the fingers and less severely impaired at the wrist, elbow and shoulder. There was pseudoathetosis of the right hand and, when her eyes were closed, the right arm would drift and she did not know where it was. Vibration sense was present on the right but diminished in comparison to the left. Extraocular movements were normal, the visual fields were intact and there was no visual or tactile extinction

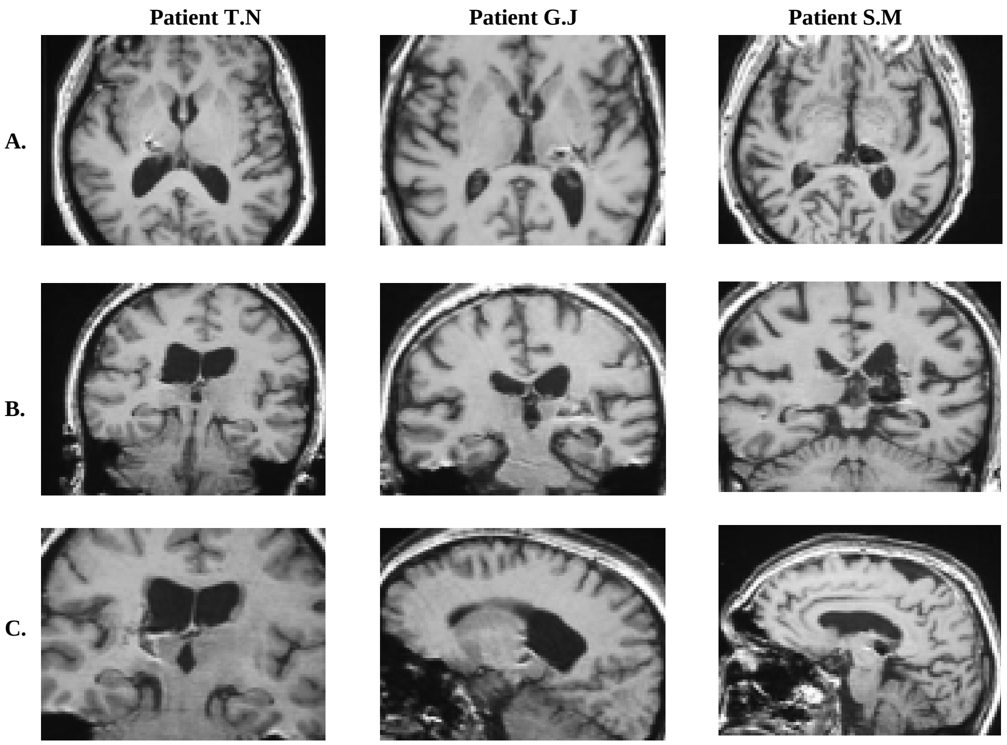

Fig. 1. Horizontal (A), Coronal (B), and Midsagittal (C) MRI scans for each of the patients. Note that the scan for patient T.N in row C is a coronal section.

on double simultaneous stimulation. She was able to walk with a cane.

G.J is a 54 year old right handed man who suffered a haematoma centered in the left thalamus in January 2000. Examination at the time of testing 3 months later showed only slight weakness of the right hand and arm and increased stretch reflexes in the right fingers and wrist. He was able to oppose his right thumb to the 5th finger. There was dense sensory loss to all modalities (touch, temperature, pin, position and vibration) in the right hand; these deficits were also severe in the proximal arm and leg although with some recovery of position sense proximally. Extraocular movements were normal, the visual fields were intact and there was no visual or tactile extinction on double simultaneous stimulation. He was able to walk with a cane.

S.M is a 61 year old, right handed, hypertensive man who suffered a haematoma centered in the left pulvinar in January 2000. Examination at the time of testing 2 months later revealed mild weakness of the hand, but somewhat greater weakness of the shoulder and the tendon stretch reflexes were normal. Light touch, pin and temperature sensation were intact. Stereognosis was very poor in the right hand. Position sense was impaired in the right hand, although he could determine the direction of finger movement for large excursions. Extraocular movements were normal, the visual fields were intact and there was no visual or tactile extinction on double simultaneous stimulation. He was able to walk with a cane.

### *2.1.2. Apparatus and stimuli*

Stimulus presentation and response collection were controlled by a Macintosh Powerbook 3400c. Throughout the experiments duration three black unfilled permanent boxes (the defining lines were one pixel wide) measuring 4 cm on each side were displayed along the horizontal axis on a background colored light gray. The middle of the center box was centered on a black fixation cross (font Chicago, size 12) that appeared at the beginning of a trial and was terminated by a response. The center of each peripheral box was 6 cm from that of the central box. The cue was a black unfilled square (the defining lines were three pixels wide) measuring 2.8 cm on each side that appeared centered on the middle of either the left or right permanent boxes. The target stimulus that appeared in the middle of one of the peripheral boxes was a filled black square measuring 1.3 cm on each side. The stimuli and a representative trial are shown in Fig. 2.

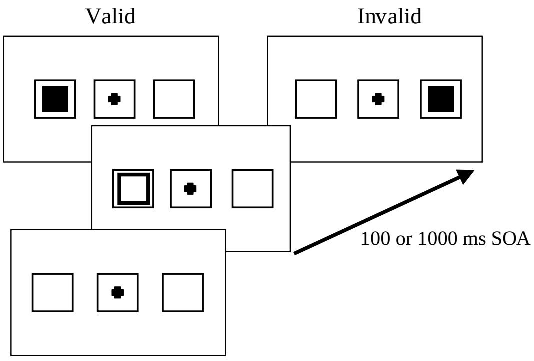

Fig. 2. A typical trials sequence for Experiment 1 in which a cue appearing in the left visual field is followed by a target appearing in the right visual field.

### *2.1.3. Procedure*

Each patient completed multiple blocks of trials. In each session the computer monitor was placed at a viewing distance of approximately 80 cm. Patients were instructed to maintain fixation and as quickly and as accurately as possible to press with the index and middle finger of their ipsilesional hand the ‘v’ button for targets that appeared in the left box and the ‘n’ button for targets that appeared in the right box.

Each trial began with the fixation point appearing in the middle of the central box. After 500 ms a cue appeared for 250 ms either in the left or the right peripheral box. In the short stimulus onset asynchrony (SOA) condition the target appeared 100 ms after cue onset and disappeared together with the cue 150 ms later. In the long SOA condition the target appeared 1000 ms after cue onset and disappeared 150 ms later. Participants were provided 2500 ms to respond. The inter trial interval was 500 ms. Response latency was measured in ms and was timed from target onset to a key-press response.

Each patient completed multiple blocks of 128 trials. Within each block the cue appeared with equal likelihood to the left and right. At each SOA the target appeared 32 times at the cued location and 32 times at the non-cued location. The cue did not provide reliable information as to the targets location. Cue location, target location and SOA were each selected with equal probability, at random and combined orthogonally.

## *2.2. Results*

Only correct response trials in the range of 100 to 2500 ms that were less than 3 standard deviations from each individual’s mean were included in the analysis. Exclusion trials resulting from both errors and reaction times (RTs) outside the range accounted for less than 2% of the total number of trials.

Mean RTs were submitted to a mixed analysis of variance (ANOVA) with Cue Validity (Valid and Invalid), Target Field (Ipsilesional and Contralesional), SOA (Short and Long), and Patient (T.N, S.M and G.J) as independent variables. T.N completed 8 blocks, S.M completed 6 blocks and G.J completed 6 blocks. Each block represented a separate level of the random variable. Figure 3 shows the data for each patient.

T.N’s responses were fastest (422 ms), G.J’s responses were intermediate (855 ms) and S.M’s responses were slowest (972 ms), $F(2, 17) = 732.8$, $p < 0.005$. Replicating the findings of Rafal and Posner, (1987), RTs to contralesional targets were 56 ms shorter than to ipsilesional targets, $F(1, 17) = 74.6$, $p < 0.005$. The contralesional slowing in target detection varied across the three patients, $F(2, 17) = 21.5$, $p < 0.005$. The asymmetry in detection in the contralesional and ipsilesional fields was greatest in SM, in whom destruction of the pulvinar was most complete, and least in TN in whom the extent of pulvinar damage was the least. RTs for targets at the non-cued locations were 22 ms shorter than for targets at the cued location, $F(1, 17) = 28.9$, $p < 0.005$, and RTs at the long SOA were 38 ms shorter than at the short SOA, $F(1, 17) = 47.6$, $p < 0.005$. The effects of SOA and its interaction with cue validity were not consistent across patients, $F(2, 17) = 13.5$, $p < 0.005$. TN and GJ showed the typical effect of facilitation at the cued location at short cue-target intervals and inhibition of return (slower responses to targets at the cued location) at longer cue-target intervals; while SM showed inhibition at the cued location at both short and long intervals.

## *2.3. Discussion*

The main findings of this experiment can be summarised as follows. First, responses to contralesional targets were significantly slower than to ipsilesional targets at both SOAs whether the target appeared at the cued location or not. These data with uninformative peripheral cues extend the observations of Rafal and Posner [8] with informative peripheral cues. Moreover, the effect on contralesional target detection was proportional to the extent of pulvinar damage in these three patients. Second, IOR was found contralesionally for each of the patients. This finding can be distinguished from the lack of IOR in a single patient with unilateral damage to the superior colliculus [13]. Thus it seems that while the superior colliculus is necessary for the generation of IOR the pulvinar is not.

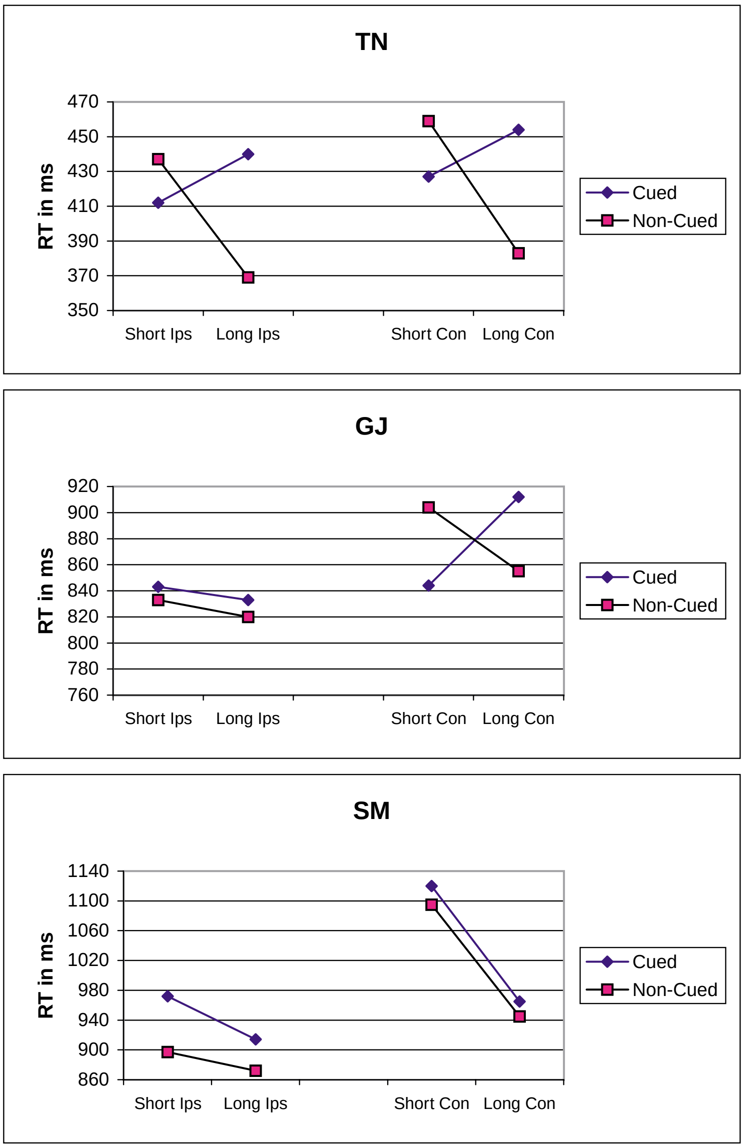

Fig. 3. Mean response times in milliseconds for each of the patients in Experiment 1. Data are shown as a function of Target Field (Contra and Ipsi), SOA (Short and Long) and Cueing Condition (Cued and Non-cued).

## 3. Series 2: Global – local interference

The putative role of the pulvinar in filtering irrelevant distractors has been addressed twice previously. In the PET study of LaBerge and Buchsbaum [7], participants identified a lateralised target that appeared either alone, or in a selective filtering task with distractors present. LaBerge and Buchsbaum found increased contralateral pulvinar activation when the contralateral visual field contained distractors relative to when it did not. From this they concluded that the pulvinar contributes to filtering of irrelevant information. To test this prediction we studied target selection in the presence of distractors in patients with unilateral pulvinar damage using a flanker interference paradigm (Danziger et al. [6]). In our experiments patients reported the colour of a square of a specified size while ignoring an irrelevant distractor that appeared either contralesional or ipsilesional to the target. The results of 2 experiments were inconsistent with LaBerge's filtering hypothesis that predicts more interference from contralesional distractors than ipsilesional distractors. First, in one experiment in which targets always appeared centrally interference effects were similar whether a distractor appeared relatively contralesional or ipsilesional to the target. And second, in an experiment in which the target appeared peripherally and was flanked by a distractor that appeared centrally interference effects were actually larger when the target appeared contralesionally and the distractor was relatively ipsilesional than when the target appeared ipsilesionally and the distractor was relatively contralesional. These results are inconsistent with LaBerge's filtering hypothesis that predicts larger interference effects when the distractor is relatively contralesional because in this case the distractor is subject to less filtering. The aim of experiment 2 was to further test the role of the pulvinar in selectivity and response activation by employing a response competition task in which participants identified a target that appeared at one level of a hierarchical figure (Navon figure) while ignoring a distractor stimulus that appeared at the remaining level of the hierarchical figure. The 'local' level of the hierarchical figure consisted of many small identical letters that were spatially arranged to form a larger letter (the global level). The stimulus set consisted of two target letters that were assigned to different responses. Stimuli at the two hierarchical levels were, with equal probability, either compatible with each other (letters at both levels are the same) or incompatible (letters at both levels are different). Typically RTs to a target are shorter when target and distractor are compatible than when they are incompatible. The presence of a RT difference between compatible and incompatible conditions indicates that the stimulus at the non-selected level is processed to the level of response.

There were three predictions. First, if the pulvinar contributes to contralesional orienting [8] then as was found in Experiment 1 responses to contralesional targets should be slower than to ipsilesional targets. Second, if pulvinar damage results in a filtering deficit then interference effects should be larger for contralesional stimuli than ipsilesional stimuli because of an inability to filter distractors. Finally, if through its reciprocal anatomical connections with the temporo-parietal junction (TPJ) the pulvinar affects TPJ processing then a deficit in attending to the global level would be expected in patients with right pulvinar lesions and a deficit in attending to the local level would be expected in patients with left pulvinar damage. This pattern would mirror the processing deficits associated with damage to the right TPJ and left TPJ respectively [14].

*3.1. methods*

*3.1.1. Subjects*

Two patients, TN with a right thalamic lesion and GJ with a left thalamic lesion, were tested in multiple blocks. Patient SM was excluded from the study as he found the task frustrating and did not wish to participate.

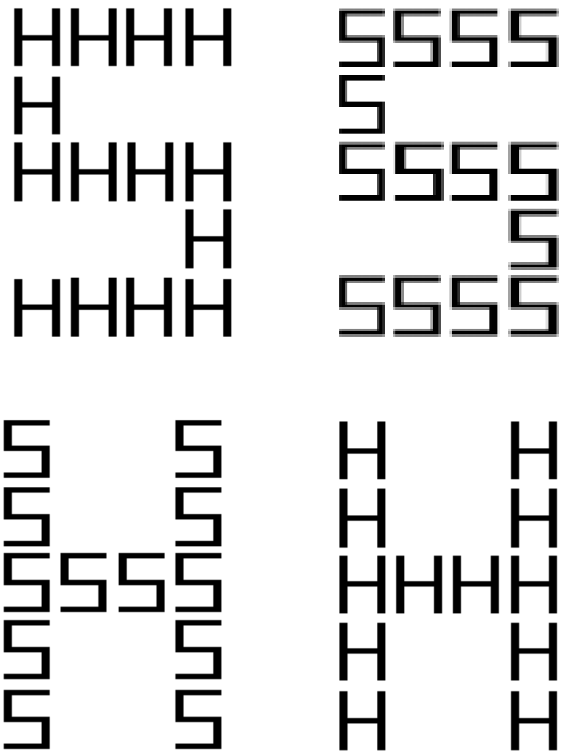

Fig. 4. The stimulus set of Experiment 2. The stimuli are not drawn to size.

*3.1.2. Apparatus and stimuli*

Stimulus presentation and response collection were controlled by a Macintosh Powerbook 3400c. The global letters (either an H or an S) were constructed by placing small letters (either an H or an S) in a $5 \times 4$ matrix. Every stimulus contained an H or an S as a target at one level of the hierarchical figure and an H or an S as a distractor at the other level. This resulted in the four combinations illustrated in Fig. 4. The global letters measured 5.2 cm in height and 3.6 cm in width and the local letters measured 0.8 cm in height and 0.6 cm in width. The inner edge of the hierarchical figure appeared 2.7 cm from fixation. The background was white and a small plus sign served as the fixation point (font Chicago, size 24).

*3.1.3. Procedure*

Each trial began with the appearance of a central fixation point. After 500 ms a hierarchical pattern appeared either to the left or right of fixation and remained visible until the patient responded or until 2500 ms had elapsed. The inter-trial interval was 1000 ms. Patients used the index and middle fingers of the ipsilesional hand to press the 'n' key when the target was an H and the 'v' key when the target was an S. Response latency was measured in ms and was timed from target onset to a key-press response.

Each patient completed multiple blocks of 80 trials alternating between blocks in which they responded to targets at the local level and those in which they responded to targets at the global level. Half of the trials in each block were response compatible and half were response incompatible. Compatible and incompatible trials were distributed equally between the visual fields. The target and distractor identity as well as stimulus field were each selected with equal probability, at random and combined orthogonally.

### 3.2. Results

The analysis included only correct response trials. Failures to respond in a speeded manner (latencies longer than 2500 ms), anticipatory responses (latencies shorter than 100 ms) and RTs greater than 3 standard deviations from each individual's mean were excluded from the analysis. For T. N these accounted for less than 1% of the trials and for G.J they accounted for less than 1% of the trials. Error rates were very low with T.N making less than 2% errors and G.J less than 3%.

The mean RTs (see Fig. 5) were submitted to two separate ANOVA's. The aim of the first analysis was to test our first two predictions. Namely, to determine whether patients were slower to respond to contralesional targets than ipsilesional targets and to explore whether contralesional interference effects were larger than ipsilesional interfererence effects. In this ANOVA the independent variables were Compatibility (Compatible and Incompatible) and Target Field (Ipsilesional and Contralesional). Block was a random variable. The aim of the second analysis was to determine whether T.N and G.J suffered selective impairments of processing local and global information, respectively. The independent variables of this ANOVA were Target Level (Global and Local), Target Location (Left and Right) and Compatibility (Compatible and Incompatible). T.N completed 6 attend local and 6 attend global blocks and G.J completed 3 attend local and 3 attend global blocks. Each block represented a separate level of the random variable. Figure 6 shows interference effects as a function of target field. Figure 7 shows interference effects as a function of target location and target level.

T.N (715 ms) responded more quickly than G.J (1269 ms), $F(1,7) = 1518,\ p < 0.001$. As is typically found with healthy observers reaction times on compatible trials (856 ms) were shorter than on incompatible trials (943 ms), $F(1,7) = 281,\ p < 0.001$. Importantly, the patients were no slower to report a contralesional target (899 ms) than an ipsilesional target (899 ms, $F < 1$) and interference effects in the two fields did not differ ($F < 1$).

The Target Level × Compatibility × Target Location interaction, $F(1,7) = 20,\ p < 0.005$, was the only additional significant effect to emerge in the second analysis. This interaction reflects the fact that in the attend global blocks interference effects were larger for stimuli presented in the right visual field (84 ms) than in the left visual field (46 ms) while for attend local blocks interference effects were larger for stimuli presented in the left visual field (138 ms) than in the right visual field (84 ms). These data indicate that in the left visual field (right hemisphere processing) global stimuli produced more interference than local stimuli whereas in the right visual field (left hemisphere processing) local stimuli produced more interference than global stimuli. These findings are consistent with previous literature in normal individuals showing a left visual field advantage for processing global stimuli and a right visual field advantage for processing local stimuli [14]. Importantly, there was no evidence for a selective impairment in global level processing for the patient with the right pulvinar lesion (T.N) or a deficit in local level processing for the patient with the left pulvinar lesion (G.J).

### 3.3. Discussion

The main finding of the present experiment was that response times to contralesional and ipsilesional targets did not significantly differ in either compatible or incompatible conditions.

One implication of this finding relates to the role of the pulvinar in engaging attention. Whereas a main effect of target field has been reliably found in spatial cueing studies that require either target detection [8] or localization (Experiment 1) it was not found in either the experiments of Danziger et al. [6] or in the present experiment that required target discrimination. At this point it is not clear whether the fact that the latter experiments required object identification or the fact that

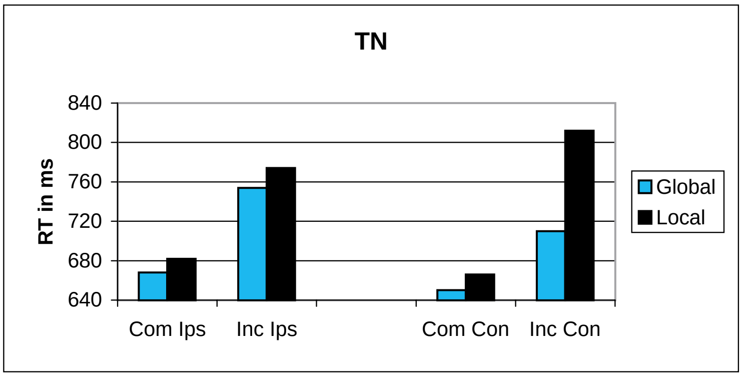

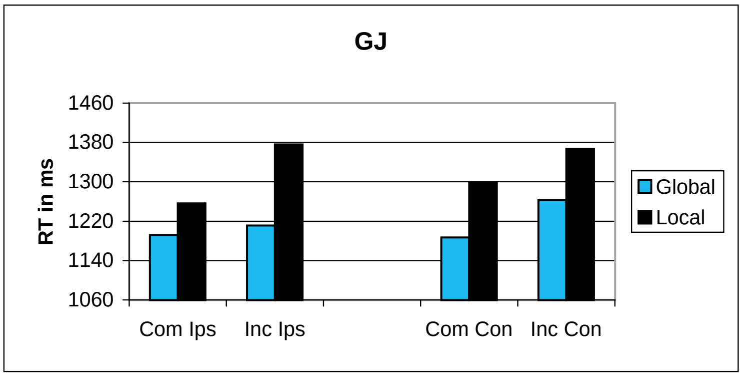

Fig. 5. Mean response times in milliseconds for each of the patients in Experiment 2. Data are shown as a function of Target Field (contra and lesi), Compatibility (compatible and incompatible) and Target Level (Global and Local).

they did not include temporally successive stimuli are responsible for this difference.[1]

A second implication relates to the hypothesis of LaBerge and Buchsbaum [7] that the pulvinar contributes to filtering distracting information. On this account pulvinar damage should hinder contralesional selection when targets and distractors compete for attention. Here we found that under such conditions response times to contralesional and ipsilesional targets did not significantly differ in either compatible or incompatible conditions. These data indicate that contralesional target selection was not impaired relative to ipsilesional target selection.

Finally, the data did not indicate that pulvinar damage affects processing of global and local information, as does TPJ damage. Whereas left TPJ damage hinders local processing and right TPJ damage hinders global processing left and right pulvinar damage did not produce similar deficits.

[1] The fact that patient S.M did not participate in Experiment 2 may have contributed to the lack of a hemifield effect in this experiment. However, S.M did not show a field effect in the discrimination task used by Danziger et al. [6].

### 3.4. *Conclusion*

Theories have speculated that the pulvinar is part of a distributed network that mediates attentional processing [1,2]. Experiments aimed at characterizing the nature of the attentional deficit following pulvinar damage have provided seemingly conflicting results. While studies requiring simple target detection [8] or target localization (Experiment 1) in a spatial cueing paradigm have shown that contralesional targets are detected more slowly than ipsilesional targets, studies requiring target identification have not ([6]; Experiment 2). In addition, evidence that the pulvinar filters and attenuates distracting information (see [7]) was not obtained either in Experiment 2 of the present study or in the flanker interference task used by Danziger et al. [6] in which central distractors that were relatively contralesional to a peripheral target produced weaker response activation than relatively ipsilesional distractors.

We previously speculated that if target and distractor processing were determined by their location relative to the focus of attention and not necessarily by their field of appearance [6] then one could account for the dif-

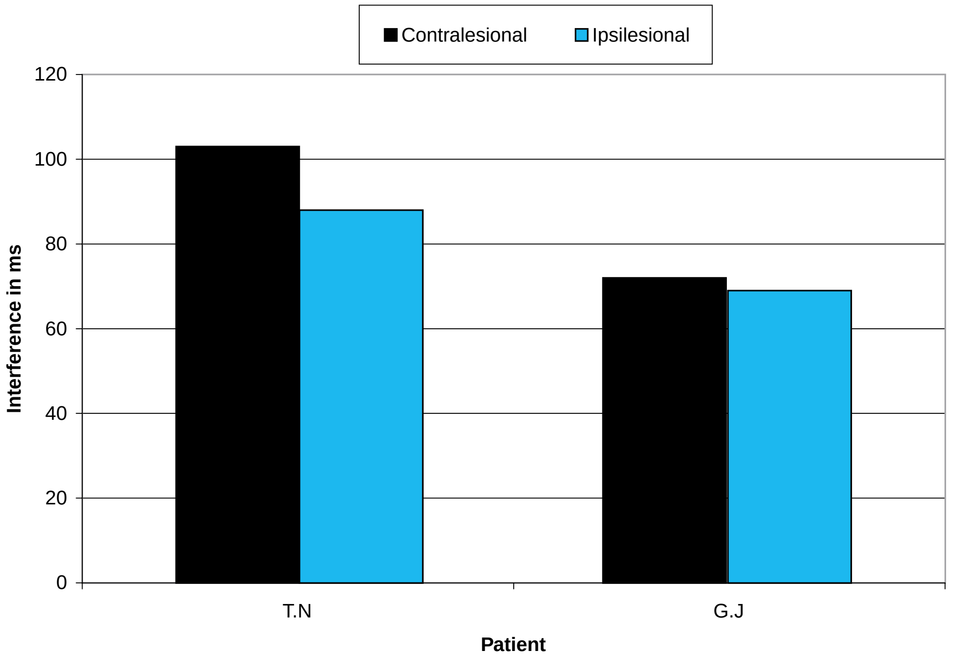

Fig. 6. Interference effects (congruent – incongruent RTs) in ms for each of the patients in Experiment 2. Data are shown as a function of Target Field (Contra and Ipsi).

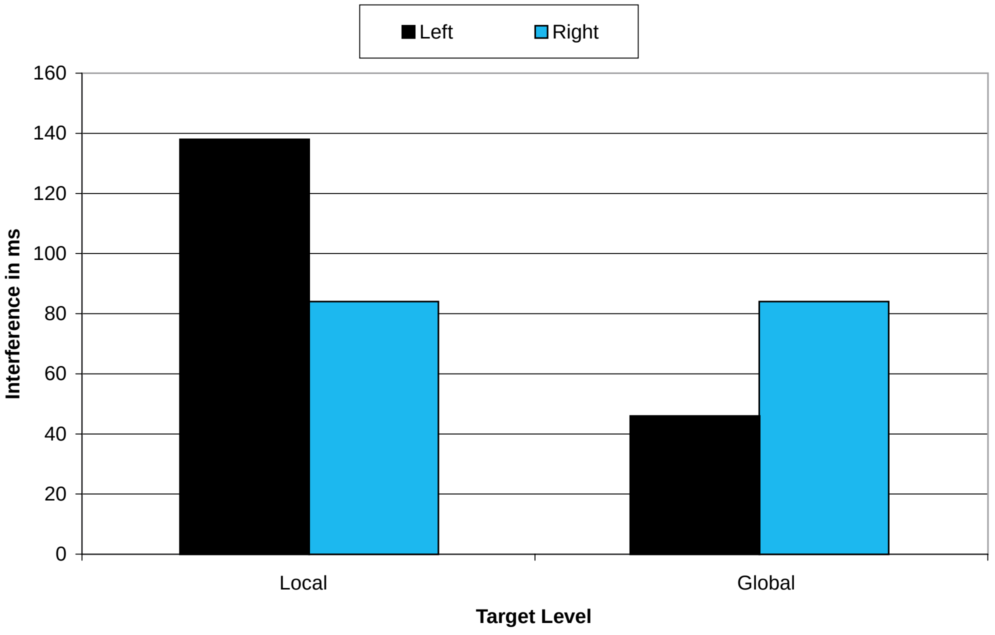

Fig. 7. Interference effects (congruent – incongruent RTs) in milliseconds for Experiment 2 as a function of Target Field (Left and Right) and Target Level (Global and Local).

ferent results obtained in the studies described above. On this account, the reference frame determining contralesional and ipsilesional space is based in part on the location of attention. For simple detection and localization tasks, it may be possible to prepare a response without focal attention to the target. In such cases, processing of lateralized targets will be most influenced by contralateral brain mechanisms. For identification or other demanding tasks, it may be necessary to focus attention or the eyes on the target location. We suggest that in this case, attention serves as a frame of reference for lateralised brain activity. For example, if the attended target were fixated, target processing would be bilateral to some degree, regardless of target location. Consistent with this view asymmetries in interference from distractors were found in the flanker task of Danziger et al. [6] in which the flanker was lateralised to the target and presumably lateralised to the focus of attention, and not in the Global Local response interference task in which the distractor was not lateralised relative to the target but rather appeared at a different hierarchical level (Experiment 2).

The attention based reference frame account also predicts slowing of responses to contralesional targets only under appropriate conditions. It could be argued that in detection and localization tasks responses are made before establishing a reference frame based on target location. In this case, the target would still be encoded primarily by response activation mechanisms lateralised to both attention and fixation, and slower responses to contralesional targets might be expected as was in fact observed in the experiments of both Rafal and Posner [8] and in Experiment 1. In contrast, in identification tasks that require attention for response activation, responses to contralesional targets would not be slower than to ipsilesional targets because targets would be processed bilaterally.

In summary our observations challenge contemporary accounts of the pulvinar's contributions to filtering of irrelevant visual information and suggest that the effect of pulvinar damage on engaging attention contralesionally may be task sensitive.

## Acknowledgements

This project was supported by an MRC grant entitled "Contributions of the pulvinar to a distributed network for selective attention" to S. Danziger and R. Ward. We sincerely thank the patients for agreeing to participate in our study.
